# O_2_ supplementation to secure the near-infrared spectroscopy determined brain and muscle oxygenation in vascular surgical patients: a presentation of 100 cases

**DOI:** 10.3389/fphys.2014.00066

**Published:** 2014-02-25

**Authors:** Kim Z. Rokamp, Niels H. Secher, Jonas Eiberg, Lars Lønn, Henning B. Nielsen

**Affiliations:** ^1^Departments of Anesthesia, Rigshospitalet, University of CopenhagenCopenhagen, Denmark; ^2^Vascular Surgery, Rigshospitalet, University of CopenhagenCopenhagen, Denmark; ^3^Interventional Radiology, Rigshospitalet, University of CopenhagenCopenhagen, Denmark

**Keywords:** blood pressure, cardiac output, cerebral oxygenation, muscle oxygenation

## Abstract

This study addresses three questions for securing tissue oxygenation in brain (rScO_2_) and muscle (SmO_2_) for 100 patients (age 71 ± 6 years; mean ± SD) undergoing vascular surgery: (i) Does preoxygenation (inhaling 100% oxygen before anesthesia) increase tissue oxygenation, (ii) Does inhalation of 70% oxygen during surgery prevent a critical reduction in rScO_2_ (<50%), and (iii) is a decrease in rScO_2_ and/or SmO_2_ related to reduced blood pressure and/or cardiac output?Intravenous anesthesia was provided to all patients and the intraoperative inspired oxygen fraction was set to 0.70 while tissue oxygenation was determined by INVOS 5100C. Preoxygenation increased rScO_2_ (from 65 ± 8 to 72 ± 9%; *P* < 0.05) and SmO_2_ (from 75 ± 9 to 78 ± 9%; *P* < 0.05) and during surgery rScO_2_ and SmO_2_ were maintained at the baseline level in most patients. Following anesthesia and tracheal intubation an eventual change in rScO_2_ correlated to cardiac output and cardiac stroke volume (coefficient of contingence = 0.36; *P* = 0.0003) rather to a change in mean arterial pressure and for five patients rScO_2_ was reduced to below 50%. We conclude that (i) increased oxygen delivery enhances tissue oxygenation, (ii) oxygen supports tissue oxygenation but does not prevent a critical reduction in cerebral oxygenation sufficiently, and (iii) an eventual decrease in tissue oxygenation seems related to a reduction in cardiac output rather than to hypotension.

## Introduction

Monitoring regional cerebral oxygenation (rScO_2_) by near infrared spectroscopy (NIRS) is used for both cardiac and non-cardiac surgery (Casati et al., 2005; Murkin and Arango, 2009) and suggested as an index for how well the circulation is managed (Murkin, 2011). A decrease in intraoperative rScO_2_ to less than 80% of the preoperative value, or to a level lower than 50% have been associated with postoperative complications such as cognitive dysfunction (Casati et al., 2005; Slater et al., 2009), stroke (Olsson and Thelin, 2006) and increased length of stay in hospital (Casati et al., 2005). Furthermore, patients with a preoperative rScO_2_ below 50% demonstrate increased probability for 1-year postoperative mortality (Heringlake et al., 2011). Thus, it appears to be an advantage to maintain rScO_2_ during surgery but with induction of general anesthesia subsequent reduction in blood pressure may affect regional blood flow and in turn tissue oxygenation (Petrozza, 1990). Yet, in patients undergoing minor surgery such as mastectomy, thyroidectomy or parathyroidectomy, a low blood pressure does not appear to affect rScO_2_ as determined by NIRS (Nissen et al., 2009). During surgery mean arterial pressure (MAP) is maintained often above 60 mmHg that is considered to represent the level that secures cerebral autoregulation (Paulson et al., 1990). On the other hand, during certain types of surgery deliberate reduction of MAP to below 60 mmHg may be initiated to limit hemorrhage (Martin and Galliano, 1965; Beaussier et al., 2000; Boonmak et al., 2013).

Patients undergoing vascular surgery are supplemented with O_2_ to prevent arterial desaturation (Dixon et al., 2005) and a high intraoperative inspired O_2_ fraction has the potential to improve postoperative outcome (Niinikoski, 1969; Hunt and Pai, 1972; Greif et al., 2000; Fries et al., 2005; Turtiainen et al., 2011). Raised inspired O_2_ fraction might affect regional blood flow to the brain (Nielsen et al., 1999; Smith et al., 2012) and skeletal muscle (Welch et al., 1977; Pedersen et al., 1999) and we aimed to assess influence of O_2_ supplementation on rScO_2_ and muscle oxygenation (SmO_2_) in a cohort of vascular surgical patients. It was addressed whether (i) preoxygenation (inhaling 100% oxygen before anesthesia) increases tissue oxygenation, (ii) inhalation of 70% oxygen during surgery prevents a critical reduction in ScO_2_ to below 50%, and (iii) a decrease in rScO_2_ and/or SmO_2_ is related to reduced blood pressure or cardiac output (CO).

## Materials and methods

Using a non-randomized single-center retrospective study-design we included vascular surgical patients enrolled in a cohort as approved by the Danish Data Protection Agency (2009-41-3617) and by the local ethical committee (H-4-2012-FSP). The evaluation included, arbitrarily, 100 patients (71 males; age 71 ± 6 years, height 171 ± 12 cm, weight 75 ± 16 kg; mean ± SD) in whom vascular surgery was performed between March 2009 and August 2011. Patients were planned for open (*n* = 23) or endovascular aortic repair (EVAR) (*n* = 56) of an abdominal aortic aneurysm, lower limb by-pass surgery (*n* = 6), an iliaco-femoral (*n* = 9) or axillo-femoral bypass (*n* = 1), open surgery for arterial mesenteric stenosis (*n* = 2), or EVAR of a thoracic aortic aneurysm (*n* = 3). Fifty-nine patients were in treatment for arterial hypertension and medication included an ACE antagonist (*n* = 36), adrenergic β-receptor blockade (*n* = 23), a calcium channel inhibitor (*n* = 18), and diuretics (*n* = 25). Ten patients were diabetics and for 14 patients suffered from chronic obstructive lung disease.

The patients were exposed to at least 6 h of fast and orally intake of clear fluids was stopped 2 h before surgery. Three-lead electrocardiography monitored heart rate (HR) and pulse oximetry assessed arterial hemoglobin O_2_ saturation (SpO_2_). A peripheral vein was used for administration of fluid and anesthetics. In accordance to local guidelines, a radial artery catheter (20 gauge; 1.1 mm) was, after local anesthesia, inserted in the arm with the highest non-invasively determined systolic blood pressure. The catheter was kept patent by isotonic saline (3 ml/h) through to a transducer (Edwards Life Sciences, Irving, CA, USA) positioned at the level of the heart. A two channel cerebral oximeter (INVOS 5100C, Somanetics, Troy, MI, USA) was used to detect rScO_2_ and SmO_2_. The reported values are taken to represent hemoglobin oxygen saturation in the tissue beneath the sensor as the ratio between deoxygenated hemoglobin and the sum of deoxygenated and oxygenated hemoglobin. Thus, as approved by the US Food and Drug Administration (510k-080769), the INVOS 5100C- determined rScO_2_ is considered a trend monitor of the hemoglobin O_2_ saturation for skin, scalp, and cortical tissue. With the NIRS-probe applied to the forehead it is assumed that capillaries within the frontal lobe contribute most to light absorbance (Madsen and Secher, 1999) but the skin, subcutaneous tissue and the scalp also contribute to change the INVOS-determined rScO_2_ (Davie and Grocott, 2012; Soerensen et al., 2012). The rScO_2_ was determined with a sensor attached to the forehead as least 2 cm above the eyebrows and that position is considered to limit an influence from the frontal sinus on rScO_2_ (Tubbs et al., 2002). Monitoring a change in NIRS-determined SmO_2_ indicates an early warning of an acute blood loss (Madsen et al., 1995) but the decision to apply a NIRS sensor to the middle part of the right biceps muscle was made by the anesthesiologist in charge and SmO_2_ is therefore reported for only 61 patients. The SmO_2_ value reflects both hemoglobin/oxyhemoglobin and myoglobin (Madsen and Secher, 1999).

Modelflow methodology (Nexfin, bmeye B.V, Amsterdam, The Netherlands) (Bogert and van Lieshout, 2005) was used to assess CO and cardiac stroke volume (SV) from the pressure curve and heart rate (HR) and MAP were monitored through the arterial line. Neuromuscular blockade was evaluated with “train of four” (Organon Dublin, Ireland). Lactated Ringer and Macrodex (Fresenius Kabi, Bad Homburg, Germany) were administered to support the central blood volume according to a goal-directed strategy (Bundgaard-Nielsen et al., 2007) as guided by SV and CO and by central venous O_2_ hemoglobin saturation in patients instrumented with a central venous catheter (via the internal jugular vein as guided by an ultrasound image). Administration of red blood cells was initiated in bleeding patients when hemoglobin was below 6 mmol/L.

The patients received no sedating drugs and in accordance to local guidelines inhalation of O_2_ was introduced using a bilateral nasal catheter. Thereafter a facial mask was applied for continued O_2_ breathing until anesthesia was induced with propofol (1 mg/kg) and fentanyl (1 μ g/kg). Cisatracurium (0.1–0.15 mg/kg) facilitated oral tracheal intubation and anesthesia was maintained with propofol (0.08 mg/kg/min) and remifentanil (0.3–0.4 μ g/kg/min). For ventilation a Dräger CATO (M32040, Lübeck, Germany) in volume-controlled mode was adjusted to an end-tidal CO_2_ tension of 4–4.5 kPa and a positive end-expiratory pressure of 5 cm H_2_O was used. When the patient was intubated, the inspiratory O_2_ fraction was set to 0.7 for maintenance of tissue oxygenation whereby the incidence of surgical site infections may decrease (Greif et al., 2000; Turtiainen et al., 2011). In 16 patients arterial blood was obtained for immediate blood gas analysis (ABL 725; Radiometer, Copenhagen, Denmark) to secure that changes in SpO_2_ reflected those in SaO_2_.

Values were recorded: (a) with the patient breathing room air, (b) after breathing O_2_ enriched air, (c) following induction of anesthesia, and finally (d) after tracheal intubation. Reported values during surgery represent the lowest noted rScO_2_ with the associated values for SmO_2_, HR, SV, CO, and MAP.

### Statistics

For normally distributed data, One-Way analysis of variance (ANOVA) with repeated measures was used. In the case of a significant main effect, a Tukey-test based *post-hoc* evaluation was applied. Correlations among variables were evaluated by Spearman's test. A GLM matrix analysis was use to locate the factor that had the statistically strongest influence on rScO_2_. A *P*-value < 0.05 was considered statistical significant.

## Results

**Figure 1:**
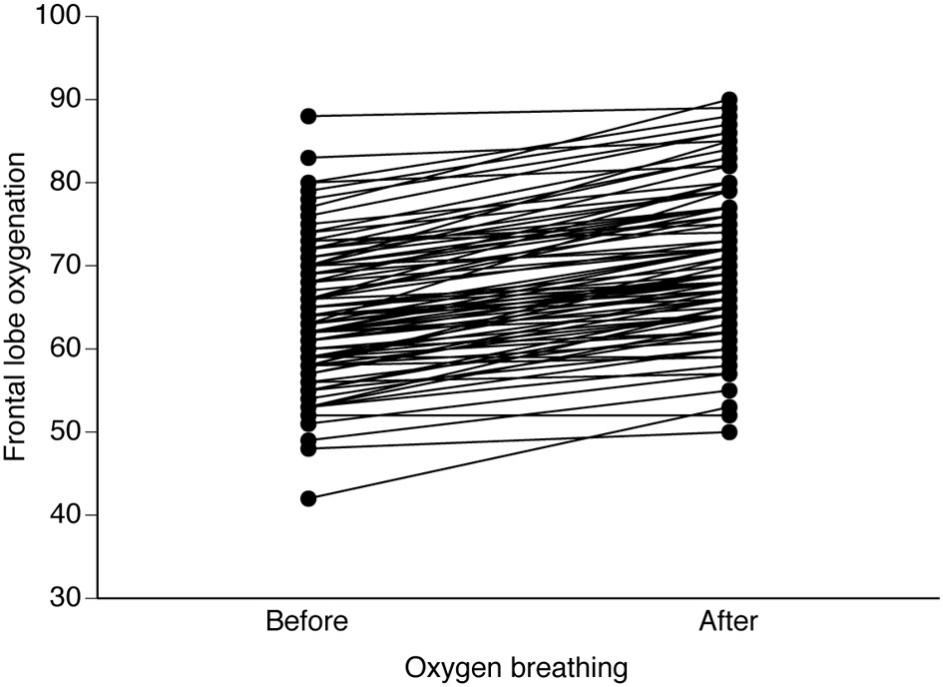
**Effects of O_2_ breathing on NIRS determined frontal lobe oxygenation.** Data are individual responses from vascular surgical patients exposed to preoperative facial mask breathing with 100% O_2_.

Breathing O_2_ enriched air increased arterial O_2_ tension (from 10 ± 2 to 34 ± 12 kPa), SpO_2_ (95.3 ± 2.4 to 99.7 ± 1.1%), arterial hemoglobin O_2_ saturation (from 96.2 ± 2.0 to 99.7 ± 0.2%), and the arterial CO_2_ tension (from 5.1 ± 0.6 to 5.4 ± 0.7 kPa; all *P* < 0.05). In all patients O_2_ breathing increased rScO_2_ (Figure 1) and SmO_2_ while there was no effect on cardiovascular variables (Table 1). Statistically, SpO_2_ contributed most to rScO_2_ (*P* < 0.0001): changes in tissue oxygenation correlated to those in SpO_2_ (rScO_2_, *r* = 0.50; SmO_2_, *r* = 43. *P* < 0.05) as provoked by breathing O_2_ enriched air before anesthesia.

**Table 1:** **Cardiovascular and blood gas variables for vascular surgical patients**.

	**Before anesthesia**		**During anesthesia**		
	**−O_2_**	**+O_2_**	**Induction**	**Intubation**	**Surgery**
SpO_2_ (%)	95.3 ± 2.4	99.7 ± 1.1^*^	99.8 ± 1.1^*^	99.8 ± 0.9^*^	99.9 ± 0.2^*^
ScO_2_ (%)	65 ± 8	72 ± 9^*^	70 ± 10^*^	75 ± 8^*^	66 ± 7^†^
SmO_2_ (%)	75 ± 9	78 ± 9^*^	80 ± 7^*^	79 ± 7^*^	80 ± 8^*^
HR (b min^−1^)	73 ± 13	72 ± 14	65 ± 13^*^	72 ± 16	61 ± 11^*^^†^
MAP (mmHg)	103 ± 18	102 ± 18	69 ± 18^*^	86 ± 25^*^	64 ± 11^*^^†^
SV (ml)	67 ± 16	66 ± 16	60 ± 17^*^	59 ± 17^*^	69 ± 15^†^
CO (L min^−1^)	4.9 ± 1.3	4.8 ± 1.3	4.1 ± 1.2^*^	4.3 ± 1.3^*^	4.1 ± 1.1^*^

Values are means ± SD with (+O_2_) and without (−O_2_) preoxygenation and following induction of anesthesia, immediately after the patient was intubated, and during surgery 47 ± 24 min after intubation. CO, cardiac output; HR, heart rate; MAP, mean arterial pressure; SV, cardiac stroke volume; SpO_2_, puls oximetry determined hemoglobin O_2_ saturation in arterial blood; SmO_2_ and ScO_2_, near infrared determined muscle and frontal lobe oxygenation, respectively.

* Different from before anesthesia without O_2_ supplementation.

† Different between intubation and surgery; P < 0.05.

**Figure 2:**
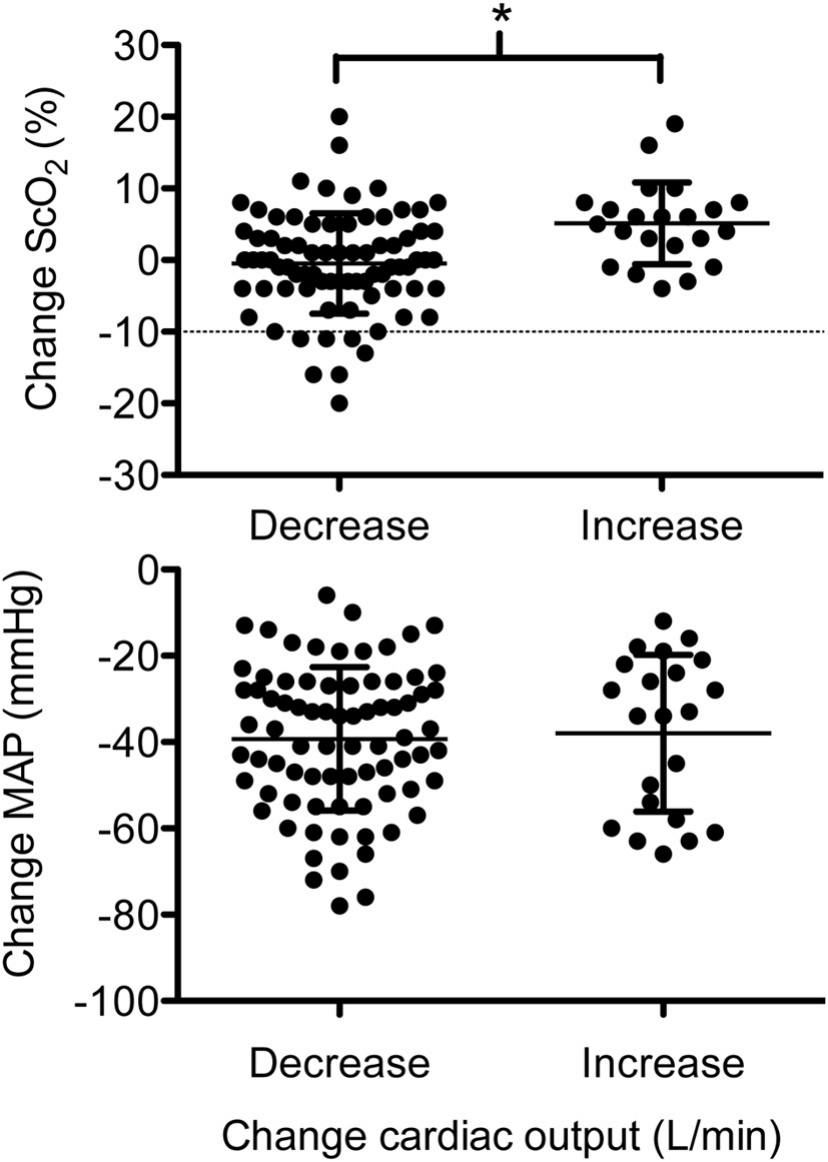
**The surgical changes in frontal lobe oxygenation (ScO_2_) and mean arterial pressure (MAP) related to cardiac output (CO; decrease is CO below the preoperative level and increase is CO above the preoperative level).** The dotted line straight line (upper panel) at −10% represents the change in ScO_2_ considered to be critical. ^*^Different value; *P* < 0.05.

Following induction of anesthesia, MAP, HR, and CO lowered, while rScO_2_ and SmO_2_ remained elevated. For three patients rScO_2_ was reduced by more than 10% indicative of a potentially critical reduction in regional cerebral O_2_ supply. During surgery with an end-tidal CO_2_ pressure of 4.4 ± 0.4 kPa, SV, and CO were at the levels before anesthesia, SmO_2_ remained elevated and rScO_2_ was not significantly changed as compared to the preoperative level. In seven patients, however, rScO_2_ decreased by more than 10% and for five of these patients rScO_2_ was below 50%. In the patients demonstrating a significant drop in rScO_2_, CO was reduced by 1.6 ± 1.2 L/min but rScO_2_ appeared independent of MAP when CO was maintained or increased (Figure 2). Thus, there was no statistical significant relation between rScO_2_ and MAP but ScO_2_ was correlated to SV and CO (Table 2). Also rScO_2_ correlated to age, while SmO_2_ correlated only to SV and CO before but not after induction of anesthesia (*P* = 0.0025).

**Table 2:** **Relationship between frontal lobe oxygenation and cardiovascular variables**.

	**Breathing atm**	**Breathing O_2_**	**Anesthesia**	**Intubation**	**Surgery**
HR	−0.02/*P* = 0.8529	0.01/*P* = 0.9451	0.02/*P* = 0.8689	0.01/*P* = 0.8987	−0.06/*P* = 0.5814
MAP	−0.01/*P* = 0.9258	−0.01/*P* = 0.9532	0.21/*P* = 0.8689	0.11/*P* = 0.2565	0.11/*P* = 0.2917
SV	0.36/*P* = 0.0002^*^	0.34/*P* = 0.0006^*^	0.23/*P* = 0.0392^*^	0.29/*P* = 0.0032^*^	0.30/*P* = 0.0724
CO	0.40/*P* < 0.0001^*^	0.40/*P* < 0.0001^*^	0.32/*P* = 0.0013^*^	0.36/*P* = 0.0003^*^	0.20/*P* = 0.0513
SpO_2_	0.10/*P* = 0.3433	0.07/*P* = 0.5195	0.02/*P* = 0.8116	0.04/*P* = 0.7232	0.04/*P* = 0.7262
Age	−0.20/*P* = 0.0415^*^	−0.19/*P* = 0.0599	−0.18/*P* = 0.0697	−0.20/*P* = 0.0420^*^	0.09/*P* = 0.3985
FiO_2_					0.16/*P* = 0.1133
CO_2_					−0.16/*P* = 0.1183

Correlations are evaluated by Spearmans Rank Test and the included numbers are the coefficient of contingence with its level of statistical significance as determined by two-tailed t-test. CO, cardiac output; FiO_2_, inspired O_2_ fraction; HR, heart rate; MAP, mean arterial pressure; SV, cardiac stroke volume; SpO_2_, pulse oximetry determined hemoglobin O_2_ saturation in arterial blood.

* Marks the variable with statistical significance at P < 0.05.

## Discussion

This study aimed to answer three questions: (i) Does preoxygenation (inhaling 100% oxygen before anesthesia) increase tissue oxygenation? (ii) Does inhalation of 70% oxygen during surgery prevent a reported critical reduction in ScO_2_ to 50%? and (iii) When ScO_2_ and/or SmO_2_ decrease, is the decrease then related to reduced blood pressure and/or cardiac output? In vascular surgical patients, administration of elevated inspiratory O_2_ fraction increased oxygenation of both the cerebral frontal lobe (rScO_2_) and skeletal muscle (SmO_2_) (by 10 and 3%, respectively). Importantly, this increase in tissue oxygenation appeared protective for development of tissue hypoxemia following induction of anesthesia, although the incidence of critical reduction in rScO_2_ remained 5%. The third important observation was that during anesthesia a correlation between tissue oxygenation and a decrease in MAP was not observed indicating that for vascular surgical patients, as for patients scheduled for other types of surgery (Nissen et al., 2009), a transient drop in blood pressure to below what is often considered the lower limit of cerebral autoregulation does not affect rScO_2_. On the other hand, rScO_2_ correlated to a reduction in CO and SV.

It has not been evaluated whether it is profitable to control flow-related variables (SV, CO, or SvO_2_) in conjunction with an effort to maintain rScO_2_ during surgery. With fluid administration according to an “individualized goal-directed regime,” SV and hence CO is optimized to a level considered to represent normovolemia (Bundgaard-Nielsen et al., 2010). In the present study, the cardiovascular variables reported during surgery represent situations where rScO_2_ reached a minimum and the associated CO may reflect that fluid resuscitation was about to be initiated.

A correlation between rScO_2_ and CO supports a link to blood flow (Ide et al., 1999) and as cardiovascular capacity decline with advancing age (Proctor and Joyner, 1997), this view is further supported by a correlation between rScO_2_ and age. Seven patients suffered a critical reduction in rScO_2_ when CO dropped (Figure 2) and if O_2_ supplementation had not induced a 10% increase in rScO_2_, it is likely that rScO_2_ would have been reduced to a critical level in more patients. We did not find indication for that a low MAP affected rScO_2_ and in ASA class I patients, a 30% reduction in MAP with a minimum MAP of 50 mmHg, is considered acceptable (Yamada et al., 1988; Petrozza, 1990). In this evaluation 32 of 100 patients undergoing vascular surgery at one stage of the operation developed a MAP < 60 mmHg, apparently without affecting rScO_2_. Even when MAP was below 50 mmHg (*n* = 12), rScO_2_ was maintained.

Administration of an O_2_ enriched atmosphere was introduced to reduce the incidence of complications after colorectal surgery (Greif et al., 2000) and vascular surgery (Turtiainen et al., 2011). Yet, not all follow-up studies support that a high O_2_ fraction reduces surgical site infections (Pryor et al., 2004; Belda et al., 2005; Meyhoff et al., 2009; Bustamante et al., 2011) and O_2_ supplementation may provoke formation of O_2_ free radicals (García-de-la-Asunción et al., 2011). Furthermore, surgical site infection, atelectasis, pneumonia, and respiratory failure occur at similar frequencies in patients with an inspired O_2_ fraction of 0.80 compared to 30% O_2_ (Meyhoff et al., 2009). The tendency for O_2_ breathing to provoke pulmonary atelectasis (Hedenstierna, 2012) suggests the use of positive end expiratory pressure as applied in this evaluation. Also the relevance for using O_2_ supplementation is likely to vary among groups of patients. For vulnerable patients a reduction in tissue oxygenation may provoke ischemic stroke after surgery (Waggoner et al., 2001; Cheng-Ching et al., 2010). Importantly, vascular surgical patients often present with coronary artery or cerebrovascular disease (Hertzer et al., 1984) and hypotension may become critical for maintained tissue oxygenation. We suggest that O_2_ supplementation is important for perioperative preservation of tissue oxygenation.

This study is limited by several factors: a retrospective design often fails to extract dynamic cardiovascular variables in patients exposed to surgery. Furthermore, for the patients included in the present cohort, the recommendation to use NIRS to guide the circulation during surgery may not have been followed and a placebo-controlled randomized design is in need. A third reservation relates to the NIRS used for interpretation of changes in tissue oxygenation as the INVOS cerebral oximeter appears to be sensitive to changes in skin blood flow (Davie and Grocott, 2012).

From this retrospective evaluation of tissue oxygenation including 100 patients undergoing vascular surgical procedures, it is concluded that O_2_ supplementation increases the NIRS-determined oxygenation of the cerebral frontal lobe and skeletal muscles. Furthermore, the data suggest that an elevated inspired oxygen fraction is not efficient to prevent a critical reduction in cerebral oxygenation since a decrease seems to be related to a reduced cardiac output.

### Conflict of interest statement

The authors declare that the research was conducted in the absence of any commercial or financial relationships that could be construed as a potential conflict of interest.
